# Imaging of pheochromocytoma and paraganglioma: moving beyond “lumpology” with SSTR, FDG and MIBG molecular imaging

**DOI:** 10.1186/1470-7330-15-S1-P49

**Published:** 2015-10-02

**Authors:** MS Hofman, D Pattison, RJ Hicks

**Affiliations:** 1 Centre for Molecular Imaging and Neuroendocrine Tumour Service, Peter MacCallum Cancer Centre, Melbourne, Australia and Department of Medicine, University of Melbourne, Australia

## Learning objectives

Cancer staging traditionally uses CT or MRI for detecting suspected malignant lesions with characterisation performed by histopathology following biopsy. In this “lumpology” paradigm, the number, size and location of lesions are used to determine prognosis and guide treatment strategies. To provide an educational exhibit to highlight the utility of molecular imaging to diagnose, stage and characterise pheochromocytoma (PCC) and paragangliomas (PGL) phenotype and guide patient management.

## Content organisation

Pictorial review of ^68^Ga-DOTATATE (GaTate), ^18^F-fluorodeoxyglucose (FDG) and ^123^/^124^I-metaiodobenzylguanidine (MIBG) imaging. Within the “lumpology” paradigm, molecular imaging performs well with a superior sensitivity and specificity compared to CT or MRI. The real strength of molecular imaging, however, is in characterizing different PCC/PGL phenotypes which can assist in identifying the underlying type of PCC/PGL with consequent management impact including selection of patients for radionuclide therapy. PCC/PGL can be broadly divided into a pseudohypoxic cluster and tumours mutations of receptor tyrosine kinase signalling. Mutations in the pseudohypoxic cluster lead to inhibition of oxidative phosphorylation and activation of glycolytic pathway via the Warburg effect leading to high sensitivity of FDG. Owing to high somatostatin receptor expression across the range of PCC/PGL, GaTate PET/CT is emerging as the single most useful modality. Positivity of MIBG is variable paralleling the varied catecholamine secretion profile of PCC/PGL. Knowledge of other patterns such as activation of brown fat on FDG or suppression of physiologic adrenal activity on GaTateare important in interpretation.

## Conclusion

Molecular imaging is valuable in diagnosing, staging, restaging and characterizing PCC/PGL. Integration of the molecular imaging phenotype into patient management is complementary to genetic testing and histopathology, and critical to the true realization of personalised medicine.

